# Spontaneous Massive Intrarenal Hemorrhage in a Patient With De Novo Ureteropelvic Junction Obstruction

**DOI:** 10.7759/cureus.14904

**Published:** 2021-05-08

**Authors:** Satyendra A Persaud, Fidel S Rampersad, Ryan Rattan, Ian Hosein

**Affiliations:** 1 Department of Clinical Surgical Sciences, The University of the West Indies, Port of Spain, TTO; 2 Department of Radiology, The University of the West Indies, Port of Spain, TTO; 3 Department of Pathology, San Fernando General Hospital, San Fernando, TTO; 4 Department of Urology, San Fernando General Hospital, San Fernando, TTO

**Keywords:** hemorrhage, spontaneous, ureteropelvic, endo urology, emergency

## Abstract

This case represents an even rarer presentation of ureteropelvic junction obstruction (UPJO), that of a spontaneous life-threatening hemorrhage into the renal pelvis of a patient with previously unknown UPJO. Unique to this patient was the emergent nature of the presentation, requiring life-saving surgery. A review of the literature follows a discussion of the case.

## Introduction

Ureteropelvic junction obstruction (UPJO) occurs in approximately one in 1500 adult patients where it may be congenital or occur as a result of acquired causes such as kidney stone or surgery [1]. It may present with back pain, pyelonephritis, hypertension, or even hematuria - cases may also be found incidentally at imaging, even at an advanced stage [2]. Few cases of spontaneous hemorrhage have been reported in patients with UPJO in the literature [3]. This case represents an even rarer presentation of UPJO - that of a spontaneous life-threatening hemorrhage into the renal pelvis of a patient with previously unknown UPJO. Unique to this patient was the emergent nature of the presentation, requiring life-saving surgery and this highlights several learning points.

## Case presentation

A 70-year-old man with severe dementia presented with a one-day history of gross painless hematuria. His family reported that he neither had urinary symptoms nor did he appear to be in discomfort. He had no history of trauma or symptoms of a urinary tract infection and was a nonsmoker. Apart from his dementia, he was in good health for his age, with no chronic illnesses. He had no history of anticoagulant or antiplatelet use. His vital signs were stable. His abdominal examination was negative and his rectal examination revealed a small benign feeling prostate. His urine was dark red with numerous clots. His initial creatinine was 1.1 ng/ml (glomerular filtration rate [GFR] 88 ml/min) and his hemoglobin was 12.4 g/dl with a hematocrit of 37%. Intravenous fluids were commenced, as was bladder irrigation. His hematuria settled within a few hours and he was discharged to be investigated as an outpatient with CT urography and cystoscopy as he became increasingly agitated in a hospital setting. At this point his vital signs were stable and his investigations were to be completed within 48 hours.

**Figure 1:**
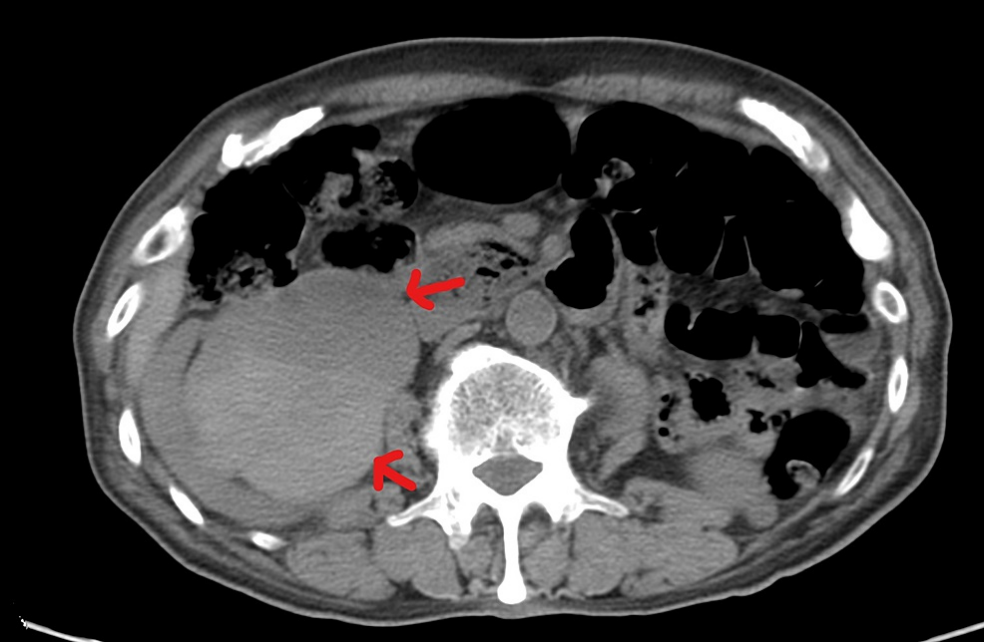
Axial CT scan through the level of the right kidney demonstrating a massively dilated right renal pelvis with large contained hematoma (red arrows).

**Figure 2:**
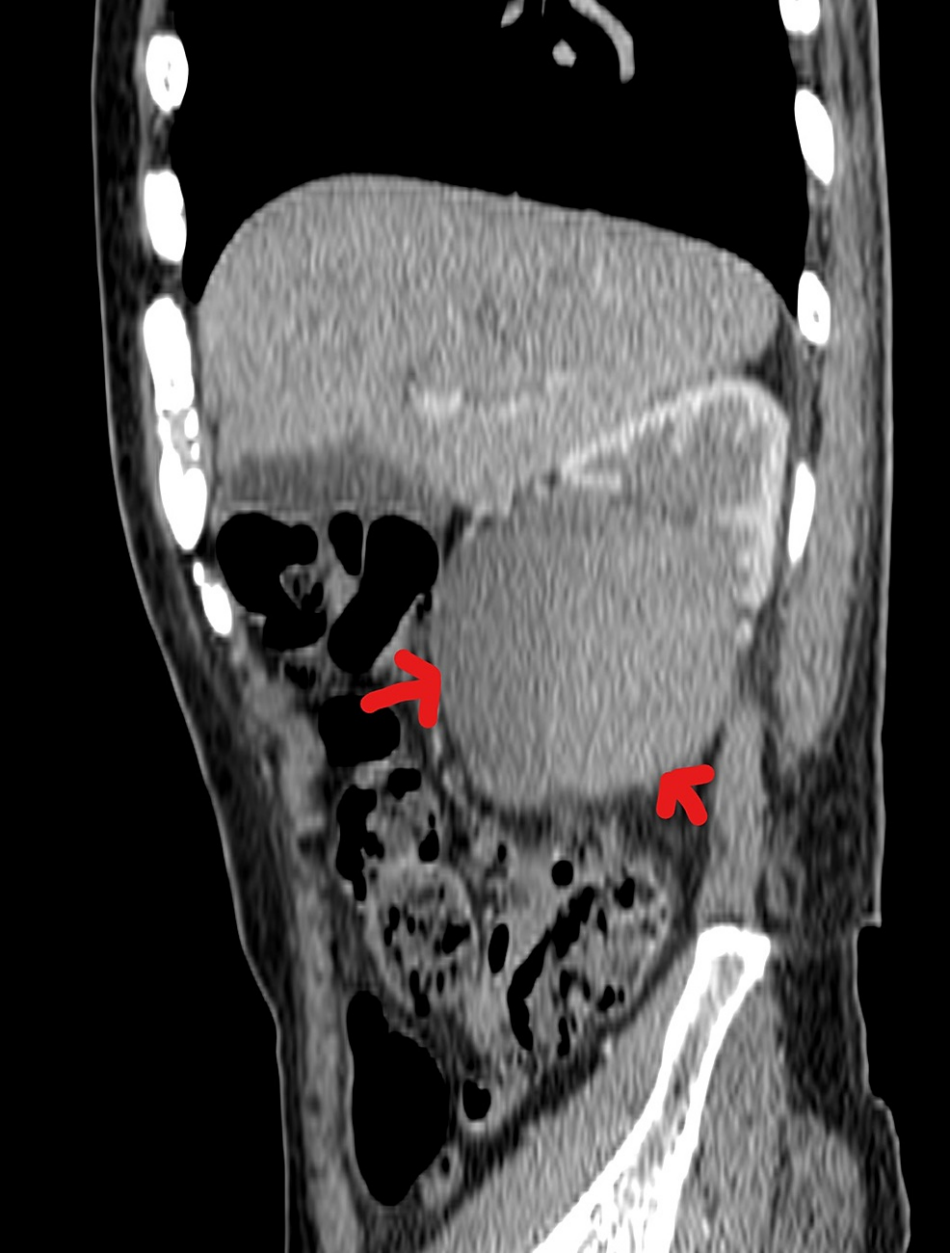
Non-contrast CT scan (sagittal view) through the level of the right kidney showing a large hematoma contained by a massively dilated renal pelvis (red arrows).

The patient represented to the ER 20 hours following discharge having been found cold and clammy at home - he was obtunded, hypotensive, and tachycardic. His relatives reported that his hematuria had recommenced and continued overnight. At presentation, his hemoglobin was noted to have dropped to 8.0 g/dl. Emergency contrast CT scan showed a grossly dilated right renal pelvis with a large contained hematoma. On imaging, there was no readily apparent cause of bleeding, nor were there any crossing vessels (Figures 1, 2).

**Figure 3:**
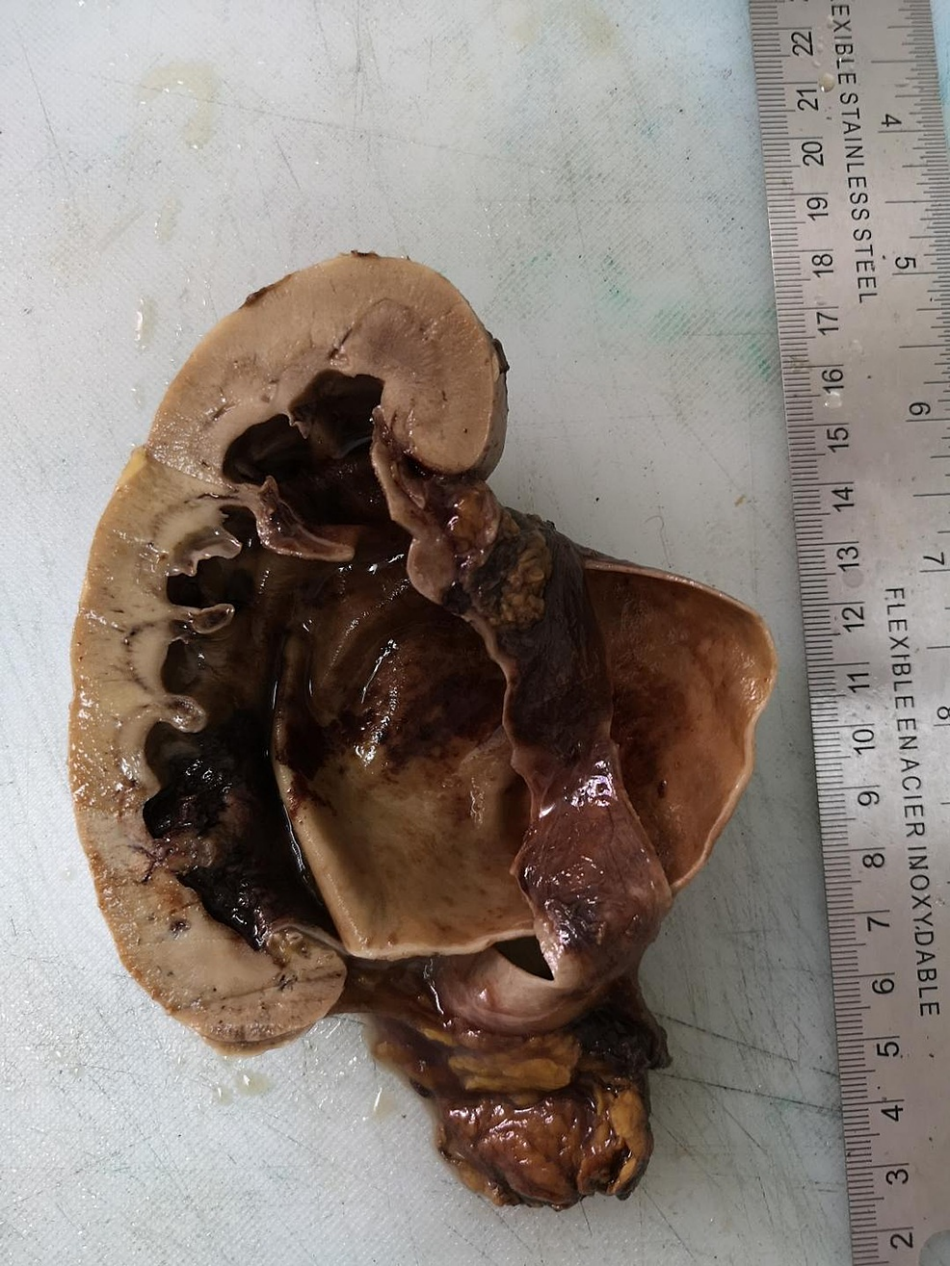
Gross section of the specimen demonstrating massively dilated renal pelvis.

The patient’s blood pressure remained labile despite fluid resuscitation and an open right nephrectomy was performed utilizing a transperitoneal, subcostal approach. Given the potential for an underlying transitional cell carcinoma, cystoscopy was carried out at the time of nephrectomy, with no intravesical lesions noted. At nephrectomy, the hematoma was contained by a grossly dilated renal pelvis and the procedure was otherwise uneventful. Pathologic examination of the excised renal unit confirmed UPJO (Figure 3).

**Figure 4:**
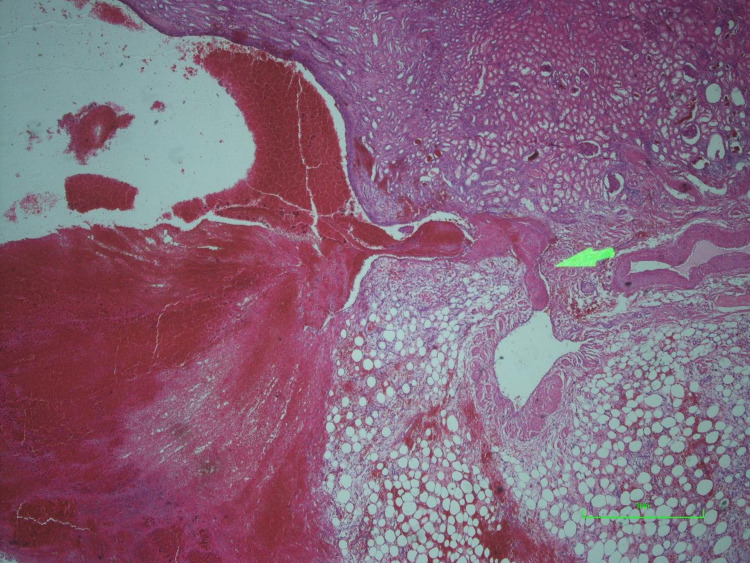
Microscopic section demonstrating rupture of a subepithelial vein into the collecting system (green arrow).

Additionally, the histological section showed no underlying malignancy or readily apparent source of the hematoma except for a large vein which ruptured into the renal pelvis (Figure 4).

The patient recovered well and was discharged on postoperative day 3. His postoperative hemoglobin count was 8.2 g/dl and he did not require any blood products. He made a full recovery and has since been followed as an outpatient. His hemoglobin count has normalized and his serum creatinine level remains within normal limits.

## Discussion

Spontaneous renal pelvic hematomas are rare with few cases reported in the literature. They have been described in normal kidneys but are more common in patients with some underlying abnormality. It is interesting that our patient had no history of any illness which would have led to ureteropelvic junction scarring. Bleeding or pelvic rupture among patients with UPJO has been described in the pediatric population following trauma [4]. There has been one reported case of spontaneous bleed in a patient with UPJO presenting as an acute abdomen [3]. While our patient did not report any pain nor appear to be in discomfort, it is unclear to what extent his dementia may have altered his expression of pain. To the best of our knowledge, this is the first recorded presentation of a spontaneous and life-threatening bleed in a patient with UPJO. In this case, the histological section revealed no underlying source of bleeding except for a vein that ruptured into the pelvis - this has not previously been described. It is unclear whether this represents one of the multiple veins which may have ruptured into the pelvis as it seems unlikely that one vein could have led to bleeding of such magnitude.

Our patient required an emergency nephrectomy. Nephrectomy was chosen due to the nature of the presentation, the potential for an underlying malignancy, and the absence of interventional radiology support. However, there are reports of alternative avenues of treatment documented in the literature. For example, Sawant et al. described drainage of a large pelvic clot using a DJ stent. Several weeks were allowed for clot resolution following which elective pyeloplasty was undertaken [3]. Lysis of clot has also been carried out using streptokinase or trypsin via a ureteral catheter [5,6]. Our patient was, however, unstable on presentation with a massive bleed and we were therefore not afforded the option of elective management.

One final learning point relates to the fact that blood loss may continue into, and be contained by a grossly dilated renal pelvis even in the absence of gross hematuria.

## Conclusions

This case highlights several learning points. Firstly, it is very rare to have spontaneous, heavy bleeding in a patient with UPJO but it should be entertained as a differential nonetheless as UPJO may be asymptomatic. Indeed, bleeding may be contained in the massively dilated pelvis, even in the absence of hematuria. Several options exist - stenting, and even lysis of clots with various agents has been described in stable patients. Finally, bleeding can be spontaneous, episodic, and massive and may even require a nephrectomy.

## References

[1] Khan F, Ahmed K, Lee N, Challacombe B, Khan MS, Dasgupta P (2014). Management of ureteropelvic junction obstruction in adults. Nat Rev Urol.

[2] Sepulveda L, Rodrigues F (2013). Giant hydronephrosis - a late diagnosis of ureteropelvic junction obstruction. World J Nephrol Urol.

[3] Sawant A, Kasat G, Pawar P, Tamhankar A (2016). Spontaneous large renal pelvis hematoma in ureteropelvic junction obstruction presenting as an acute abdomen: Rare case report. Urol Ann.

[4] Mali V, Liu B, Prabhakaran K, Loh D (2005). Blunt renal trauma in occult congenital hydronephrosis. Singapore Med J.

[5] Stegmayr B, Örsten PA (1984). Lysis of obstructive renal pelvic clots with retrograde instillation of streptokinase. A case report. Scand J Urol Nephrol.

[6] Grabe M, Forsberg B (1986). Retrograde trypsin instillation into the renal pelvis for the dissolution of obstructive blood clots. Eur Urol.

